# Nitric oxide released from JS-K induces cell death by mitotic catastrophe as part of necrosis in glioblastoma multiforme

**DOI:** 10.1038/cddis.2016.254

**Published:** 2016-09-01

**Authors:** Jessica Günzle, Nadja Osterberg, Joseph E Saavedra, Astrid Weyerbrock

**Affiliations:** 1 Department of Neurosurgery, Medical Center—University of Freiburg, Breisacher Str. 64 Freiburg, D-79106, Germany; 2 University of Freiburg, Faculty of Biology, Schaenzlestr. 1, Freiburg D-79104, Germany; 3 Cancer Inflammation Program, Center for Cancer Research, National Cancer Institute, Building 567, Room 254, Frederick MD 21702, USA

## Abstract

The nitric oxide (NO) donor JS-K is specifically activated by glutathione S-transferases (GSTs) in GST-overexpressing cells. We have shown the induction of cell death in glioblastoma multiforme (GBM) cells at high JS-K doses but the mechanism remains unclear. The aim of this study was to determine whether NO-induced cell death is triggered by induction of apoptotic or necrotic pathways. For the first time, we demonstrate that NO induces cell death via mitotic catastrophe (MC) with non-apoptotic mechanisms in GBM cells. Moreover, the level of morphological changes indicating MC correlates with increased necrosis. Therefore, we conclude that MC is the main mechanism by which GBM cells undergo cell death after treatment with JS-K associated with necrosis rather than apoptosis. In addition, we show that PARP1 is not an exclusive marker for late apoptosis but is also involved in MC. Activating an alternative way of cell death can be useful for the multimodal cancer therapy of GBM known for its strong anti-apoptotic mechanisms and drug resistance.

Glioblastoma multiforme (GBM) is the most aggressive brain cancer in humans. Despite multimodal therapies including surgery, radio- and chemotherapy the dismal prognosis of glioblastoma patients is largely caused by a prominent chemo- and radio resistance as well as an insufficient drug delivery across the blood-brain-barrier. Nitric oxide (NO), a free radical with diverse regulative functions related to immunoreactions, vascular dilatation and neurotransmission, is known for its capacity to sensitize cancer cells to radio- and chemotherapy *in vitro*.^1, 2, 3, 4^ Endogenous NO generated by eNOS or nNOS under physiological conditions is detectable in nanomolar concentrations and may act proliferative and cytoprotective and induces cell differentiation.^5^ In contrast, iNOS upon upregulation generates micromolar concentrations of NO in a variety of pathologic conditions and may induce cell death. Rigamonti *et al.* could show the upregulation of inducible NO-synthase (iNOS) after acute muscle damage by infiltration of macrophages.^6^ De Palma *et al.* observed cytoprotection in neuroblastoma cells from DNA damage by overexpression of endothelial NOS (eNOS).^7^ One explanation for this cytoprotection is the ability of NO to mediate cGMP generation and therefore the differentiation of myogenic precursor cells and prevention of apoptosis after stimulation.^8, 9, 10^ Kaczmarek *et al.* investigated the cytotoxic effect of endogenous NO in leukemia cells leading to apoptosis.^11^ This dual function of NO has to be considered when using exogenous NO released from NO oxide donors for therapeutic purposes in cancer therapy. In order to achieve an antitumour effect, micromolar doses of NO have to be delivered to the tumour cells. To stabilize the reactive and diffusing NO and to facilitate delivery of therapeutic NO doses, a prodrug was developed for *in vitro* and *in vivo* usage. The prodrug JS-K (O2-(2,4-dinitrophenyl) 1-[(4-ethoxycarbonyl)piperazin- 1-yl]diazen-1-ium-1,2-diolate) is a diazeniumdiolate that releases NO after enzymatic metabolization by glutathione S-transferases (GSTs).^12^ In previous studies we could show the specific release of NO by JS-K in GST-overexpressing GBM cells affecting their proliferation activity and viability in a dose- and time-dependent manner.^13^ *In vitro* experiments indicate the involvement of some regulatory mechanisms in a variety of tumours such as the mitogen-activated protein kinase pathways to modulate proliferation, motility and cell death.^14^ Till date it was believed that apoptosis is the major mechanism of cell death induced by NO and its derivatives. Classical apoptosis is characterized by typical morphological hallmarks including cell shrinkage and membrane blebbing. It is considered to be an active process that requires energy for protein synthesis and activation. Multiple stress-inducible molecular changes lead to the cleavage of caspases and fatal DNA damage.^15^ However, in the past necrosis has been considered to be an unregulated form of cell death.^16, 17^ That has changed since necrosis was identified to be regulated by specific molecular pathways such as the cleavage of PARP1 or when caspase-dependent pathways are inhibited.^18, 19^ Tumour cells are able to develop anti-apoptotic mechanisms implicating drug resistance. NO inhibits apoptotic mechanisms by S-nitrosylation of signalling molecules such as caspases and transcriptional factors.^20^ Apoptosis-resistant cells are monitored to bypass apoptosis by the induction of alternative cell death mechanisms like mitotic catastrophe (MC) when exposed to damaging agents.^21^ In mammalian cells MC is defined as abnormal mitosis with giant soma and multinucleated cells. Most of the tumour cells are deficient at cell cycle checkpoints and therefore deficient in reliable repair of DNA damage particularly when exposed to anticancer drugs.^22^ MC is mainly exhibited in tumour cell when exposed to chemical stress, DNA damage or chemotherapeutic agents. Authors suggest that MC is part of apoptosis and found common pathways *in vitro* such as cleavage of caspases in lung cancer cell lines or patient derived stem-like glioma cells.^22, 23^ In contrast, other groups showed that MC appears totally independent of caspase and PARP1 cleavage in leukemia *in vitro*.^24^ As shown by Castedo *et al*, cells treated with apoptosis inhibitors cannot avoid MC but the suppression of cell cycle checkpoint genes lead to MC.^25^

The main objective of this study was to investigate the molecular mechanisms of cell death induced by NO released from the diazeniumdiolate NO donor JS-K in glioma cells *in vitro*. So far, apoptosis is considered to be the major mechanism in anticancer therapy but JS-K might provide a new targeting strategy in chemoresistant cancer forms. In this context we distinguish between apoptotic and necrotic processes by alteration of the intracellular energy status, surface pattern and morphological features of cells exposed to NO and enzymatic cleavage of proteins in well-described apoptotic pathways such as caspases and PARP1.

## Results

### JS-K is cytotoxic to GBM cell lines and primary GBM cells

**Figure 1:**
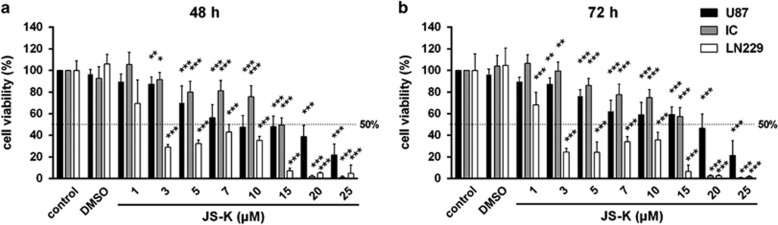
Cytotoxic effect of JS-K (1–25 *μ*M) and DMSO control (0,  48%) on U87, LN229 and primary IC cells after 48 h (**a**) and 72 h (**b**). Cell viability was determined by MTT assay at the end of the incubation time. Cytotoxicity induced by JS-K was plotted relative to viability of untreated controls set to 100% (±s.d. of three independent experiments). Asterisks (*P<0.05, **P<0.01, ***P<0.001) indicate significance between JS-K and controls

The cytotoxic effect of the NO donor JS-K was determined by MTT assay after 48 h (Figure 1a) and 72 h (Figure 1b) of treatment using concentrations between 1 and 25 μM. JS-K exhibited significant cytotoxicity (*P*<0.001) in all three GBM cell lines with an estimated IC_50_ of 2 *μ*M (LN229) or 15 *μ*M (U87, IC) for both time points. A breakdown of the viability was detectable in LN229 and IC cells at 20–25 *μ*M JS-K after 48 and 72 h. The viability of U87 cells decreased to a minimum of 21% after treatment with 25 *μ*M. These data demonstrate a dose-dependent cytotoxicity of JS-K after 48 and 72 h.

### JS-K-induced cell death is independent of the activation of caspases but leads to the inactivation of PARP1

**Figure 2:**
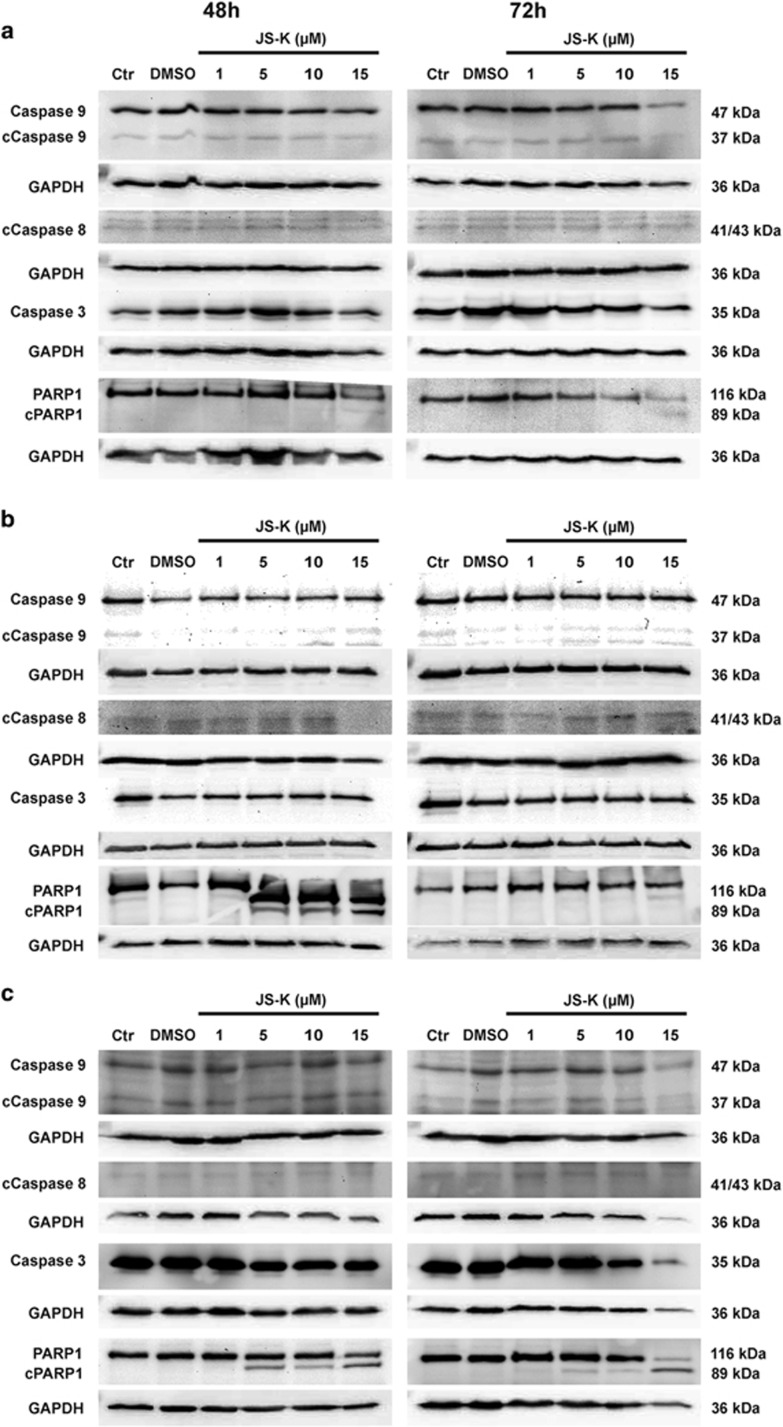
Western blot analysis for caspases 9 and 3, cleaved caspases 9 and 8 (cCaspase), PARP1 and cleaved PARP1 (cPARP1) in U87 (**a**), IC (**b**) and LN229 (**c**) cells after 48 h and 72 h exposure of JS-K (1–5 *μ*M). 25 *μ*g of protein lysates were separated with SDS-PAGE and probed with the different antibodies. Caspase 9 and 8 are expressed and cleaved equally in all three cell lines and both in untreated and DMSO controls. No cleavage and no decrease in protein level of caspase 3 can be shown. The figures shown are representative for three independent experiments

Classical apoptosis is associated with activation of initiator caspases, which are responsible for activation and cleavage of effector caspases.^26^ In this study the cleavage of the intrinsic initiator caspase 9, the extrinsic initiator caspase 8 and the effector caspase 3 was analysed by western blot in U87, LN229 and primary IC cells treated with JS-K up to 15 *μ*M for 48 and 72 h. As shown in Figure 2, procaspase 9 as well as activated caspase 9 is expressed equally in all three cell lines both in the untreated and DMSO control and in the JS-K treated cells after 48 and 72 h. For caspase 8, no increase in cleaved protein can be measured in treated cells compared to controls after 48 and 72 h. Although a constant cleavage of initiator caspases can be observed, no cleavage of effector caspase 3 can be shown. A decrease in protein level of procaspase 3 can be used as an indicator for cleavage but no change can be observed after treatment of JS-K for 48 and 72 h. These data indicate that neither classical intrinsic nor extrinsic apoptosis appears to play a role in the cytotoxicity of JS-K to GBM cells after 48 and 72 h. To confirm the role of apoptosis in JS-K treatment, the cleavage and inactivation of DNA repair enzyme PARP1 were analysed by western blot (Figure 2). U87, LN229 and primary IC cells exposed to JS-K showed a dose-dependent cleavage of PARP1 after 48 and 72 h into a fragment of 89 kDa, which is specific for apoptosis. The cleavage of PARP1 in U87 cells was observed at a concentration of 15 *μ*M of JS-K but at a concentration of 5 *μ*M in IC and LN229 cells. The fragment sizes of 40, 50 and 62 kDa, indicators for PARP1-dependent necrosis, could be observed in none of the three cell lines. Based on these results, we conclude that JS-K induced cell death is mediated in a caspase-independent way with cleavage and inactivation of PARP1 that is considered as classical apoptosis.

### JS-K prevents apoptosis by cGMP-dependent Akt-activation

**Figure 3:**
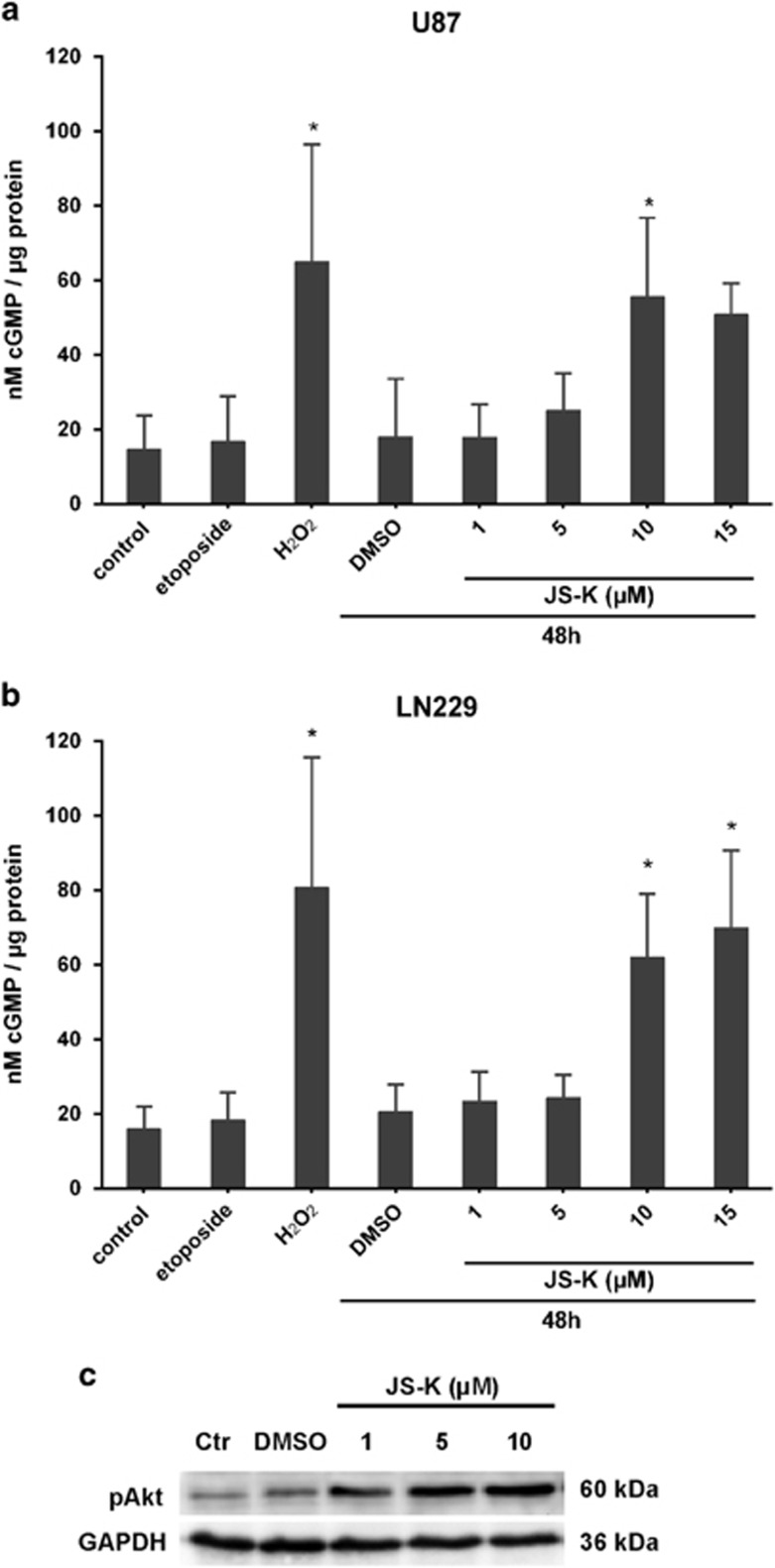
Dose-dependent increase of intracellular cGMP in U87 (**a**) and LN229 (**b**) cells mediated by JS-K after 48 h as well as in necrotic controls exposed to H_2_O_2_ (3 mM). cGMP level remained stable after induction of apoptosis by etoposide (10 *μ*M). Asterisks (*P<0.05, **P<0.01, ***P<0.001) indicate significance compared to untreated controls. Western blot analysis for pAkt in LN229 (**c**) cells show increase of phosphorylation after exposure to JS-K for 48 h compared to control. The blot is representative for three independent experiments

The secondary messenger cGMP pathway is a major mediator of the physiological effects of NO since it activates soluble guanylyl cyclase resulting in the intracellular production of cGMP.^27^ Treatment of GBM cells with JS-K for 48 h results in a dose-dependent increase of intracellular cGMP (Figures 3a and b). Cells exposed to 10 *μ*M etoposide exhibit no difference in cGMP level compared to controls whereas induction of necrosis by H_2_O_2_ revealed significant increase. From this result we suppose that the cGMP pathway is part of a necrotic cell death mechanism mediated by JS-K in GBM cells. The protein kinase Akt has been shown to suppress apoptosis and promote cell survival.^28^ Since NO is known to activate Akt-signalling via cGMP pathway we determined the phosphorylation of endogenous Akt. We examined the involvement of the Akt survival-signal by western blot with dose-dependent increase of phosphorylated Akt (Figure 3c). This demonstrates that the Akt-pathway may play an important role in the non-apoptotic cGMP-dependent effects of NO in GBM cells.

### JS-K causes a strong decline of ATP metabolism associated with necrotic mechanisms

**Figure 4:**
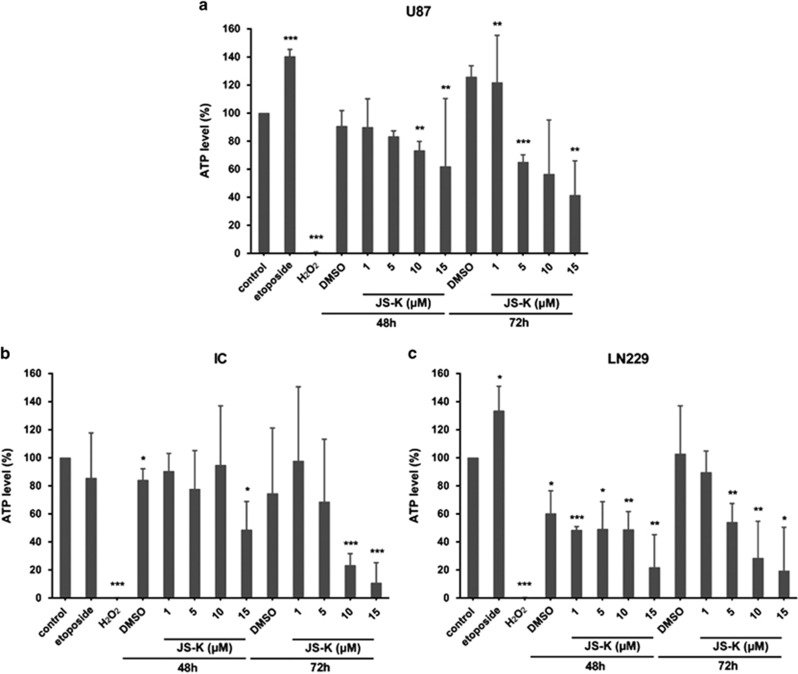
Dose- and time-dependent decrease of intracellular ATP level in U87 (**a**), IC (**b**) and LN229 (**c**) cells after exposure to JS-K for 48 and 72 h. ATP level increased after treatment with etoposide (10 *μ*M) in U87 (**a**) and LN229 (**c**) and decreased by H_2_O_2_ (3 mM) after 4 h exposure in all three cell lines. Mean values±s.d. of three independent experiments are plotted. Asterisks (*P<0.05, **P<0.01, ***P<0.001) indicate significance between JS-K and untreated controls

**Figure 5:**
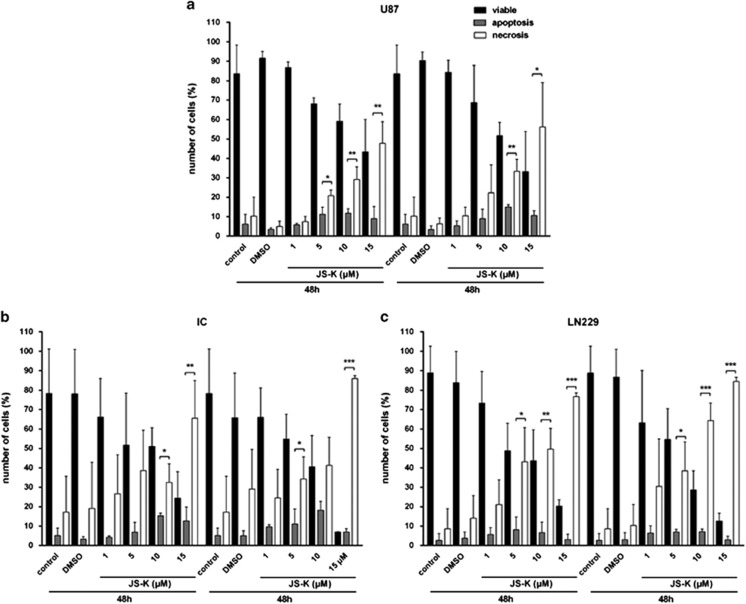
Flow cytometry analysis of annexin V and PI in U87 (**a**), IC (**b**) and LN229 (**c**) cells exposed to JS-K for 48 and 72 h and both untreated and DMSO-controls. 2 × 10^4^ cells were analysed by FACSCalibur and show significant increase of necrotic cell population. Treatment groups were normalized to total cell number and plotted relative to untreated controls±s.d. of three independent experiments. Asterisks (*P<0.05, **P<0.01, ***P<0.001) indicate significance between apoptotic and necrotic population

Adenosine triphosphate (ATP) is essential for variable cellular processes and is provided by oxidative phosphorylation in mitochondria.^26, 29^ When cells are affected by apoptosis, additional ATP is provided for protein synthesis and activation by glycolysis in the cells as demonstrated by treatment of U87 and LN229 cells with 10 *μ*M etoposide for 4 h (Figures 4a and b). Primary IC cells behaved similar to controls (Figure 4c). Necrosis is considered to be a less regulated process: the cell metabolism collapses by dysfunction of the energy conversion in the mitochondria as shown in U87, LN229 and primary IC cells by treatment with 3 mM H_2_O_2_ for 4 h (Figure 4). Cells exposed to incremental JS-K doses (1–15 *μ*M) for 48 and 72 h exhibit a dose-dependent decrease in the intracellular ATP level to 41% (U87, *P*<0.01), 11% (IC, *P*<0.001) and 19% (LN229, *P*<0.05) compared to untreated controls. The decrease in intracellular ATP caused by JS-K treatment is even stronger after 72 h, thus this effect is time- and dose-dependent. Since classical apoptosis is an energy-dependent process and as JS-K induces a breakdown of the intracellular ATP level, we associate this mechanism with a necrotic process in dying cells. To identify the amount of cells undergoing necrosis after JS-K treatment (1–15 *μ*M, 48 and 72 h), cells were stained with propidium iodide (PI) and measured by flow cytometry (Figure 5). The apoptotic cell population was discriminated from necrotic cells by annexin V staining. Both populations were compared to the amount of viable cells. As expected, in congruity with the results of the ATP assay, the population of necrotic cells increased in a dose- and time-dependent manner to 56% (Figure 5a, U87, *P*<0.01), 86% (Figure 5b, IC, *P*<0.001) and 84% (Figure 5c, LN229, *P*<0.001) upon treatment of JS-K after 72 h while the quantity of apoptotic cells remained stable over time and independent of the JS-K concentration. These results confirm our hypothesis that JS-K induces ATP-depletion in a dose- and time-dependent manner leading to necrosis after 48 and 72 h.

### JS-K induces mitotic catastrophe as part of necrosis

**Figure 6:**
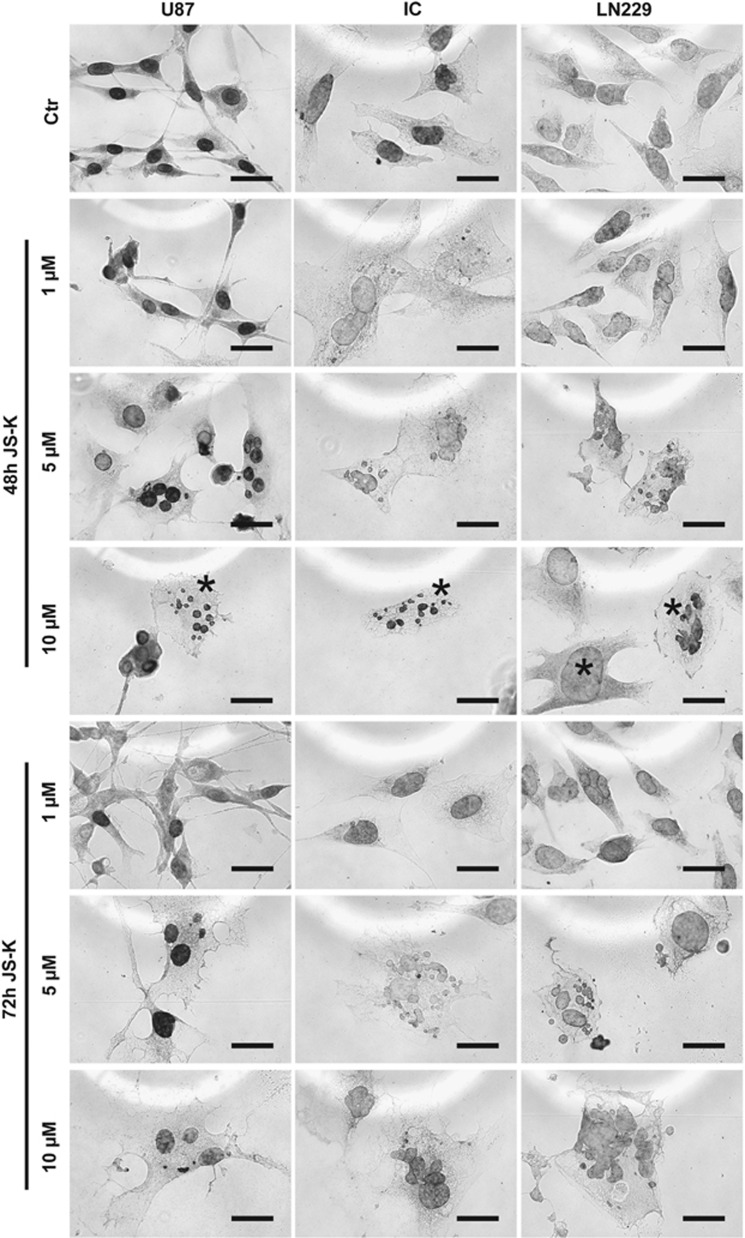
Figures show MC induced by JS-K (1–10 *μ*M) in U87, IC and LN229 cells after 48 and 72 h. Characteristics of MC are indicated with asterisk as example for 10 *μ*M JS-K after 48 h. Samples were analysed by microscopy (40 ×, scale bar represent 25 *μ*m). Data are representative for three independent experiments

**Figure 7:**
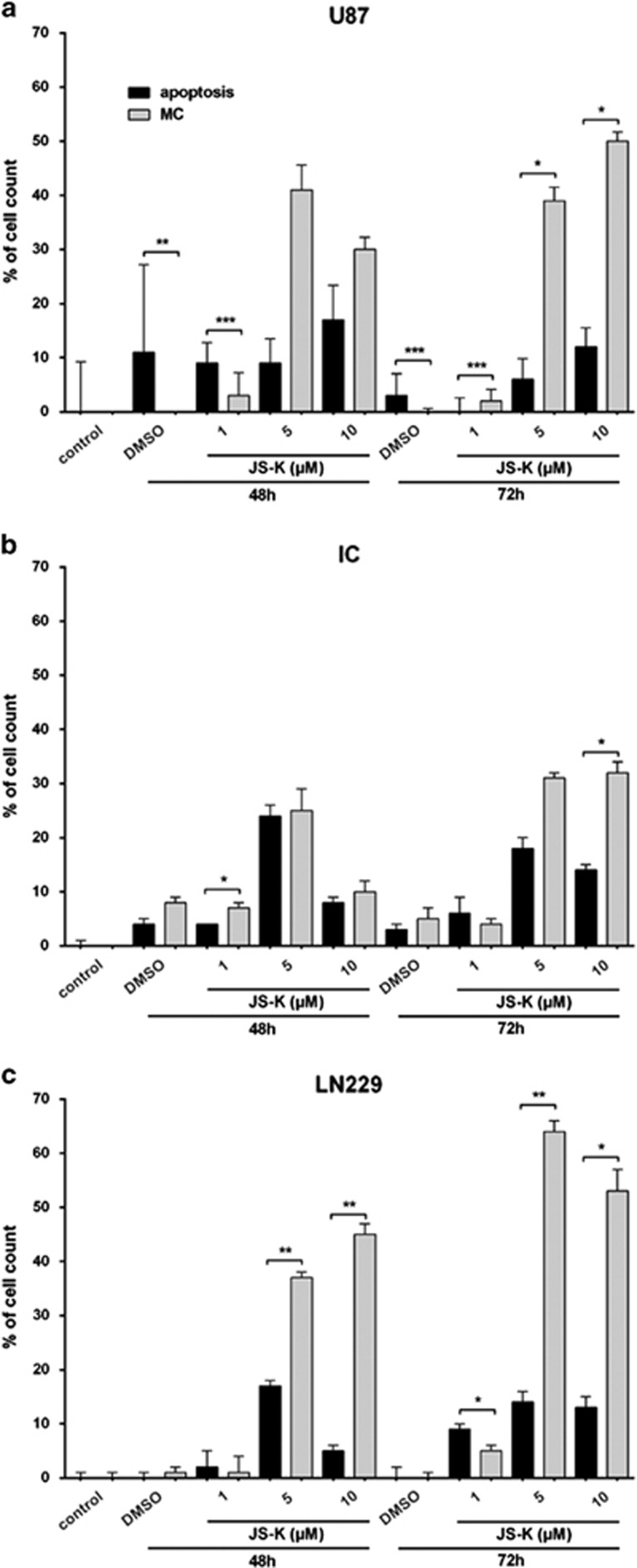
Quantitative analysis of TUNEL and MC in U87 (**a**), IC (**b**) and LN229 (**c**) cells after exposure to JS-K for 48 and 72 h. The induction of MC was determined by counting cells with characteristics described by Roninson *et al.* Induction of cell death by JS-K was plotted relative to total cell number and show a significant dose- and time-dependent increase in MC compared to apoptosis. Asterisks (*P<0.05, **P<0.01, ***P<0.001) indicate significance between apoptosis and MC with ±s.d. of three independent experiments

Mitotic catastrophe is considered to be a new form of cell death and is also obvious in JS-K treated cells. U87, LN229 and primary IC cells were exposed to JS-K (1–10 *μ*M) for 48 and 72 h and examined by microscopy (Figure 6). To distinguish apoptotic cells exhibiting fragmented DNA, a TUNEL assay was performed with the same samples. The quantitative calculation was set to the total cell number per slide and the significance refers to apoptotic cells compared to cells in MC (Figure 7). The cells showed diverse changes mainly in the nuclei. They were either multifragmented with an expanded soma or the nucleus filled a considerable part of the soma starting at a dose of 1 *μ*M JS-K in IC and LN229 and at 5 *μ*M JS-K in U87 cells (Figure 6). The number of apoptotic cells remained under 20% of the total cell number in all three cell lines and increased slightly with the concentration of JS-K compared to the untreated controls and the DMSO controls (Figure 7). In contrast, the number of cells with MC increased up to 53% of the total cell number in LN229 after 72 h and showed a dose- and time-dependent increase in all cell lines. This increase in MC is significant compared to the apoptotic population (*P*<0.05) at doses as low at 1 *μ*M JS-K. Considering these results and the findings from the flow cytometry we conclude that the size of the population with MC and the size of the population with necrosis are more or less identical. Therefore, we conclude that the phenomenon of MC must be either a part of necrosis or leads to necrosis in a separate pathway.

## Discussion

NO is known for its antiproliferative and cytotoxic effects in glioblastoma cells and the impact on migration, invasion and angiogenesis in various tumour cells in micromolar concentrations.^13, 30^ NO induces DNA double-strand breaks and post-translational modifications of proteins, for example S-nitrosylation resulting in caspase-dependent and caspase-independent pathways.^30^ JS-K is an arylating agent for molecules containing thiols. It is an NO donor prodrug that releases NO under enzymatic catalysis by GST isoforms.^31^ GSTs are overexpressed in various tumours including GBM as shown in previous experiments.^13, 32^ Thus, JS-K application in GBM cells allows a cell type-specific intracellular NO release. The impact of JS-K was described by our group as dose- and time-dependent concerning the inhibition of proliferation and induction of cell death.^13, 31^ In this study, we show for the first time that JS-K induces cell death in a dose- and time-dependent manner by MC as a new cell death mechanism or as a pre-stage of necrosis *in vitro*. Although NO is a highly diffusible and reactive molecule and rapidly reduced in cells, we could observe the strongest cellular effects in glioblastoma cells after 48 h of treatment with JS-K. Therefore, all experiments were performed after an exposure time of 48 and 72 h to investigate the strength and the duration of the NO effect. JS-K treatment was shown to decrease viability of malignant tumour cells dose-dependently at several points in time.^11, 13^ After 72 h, we could see a slight recovery in viability of U87 glioblastoma cells after JS-K exposure (5–20 *μ*M) in the MTT assay. We conclude that glioblastoma cells already acquired resistance mechanisms to repair NO-induced damages and regain the capacity for proliferation. Different tumour cells reveal variable sensitivities to JS-K, which might be attributed to resistances to NO-induced cell death mechanisms such as the expression of the detoxification enzymes GSTs.^33^ Even if there are many indications in this study that necrosis plays the major role in cell death by JS-K, apoptosis is also induced in a small population of cells what might be due to the effect of extrinsic signals by this population itself. Apoptosis is considered by several groups to be the process of cell death induced by JS-K through activation of caspases and fragmentation of DNA.^11, 34^ In case of GBM cells, it cannot be confirmed by western blot analysis that this is classical caspase-dependent apoptosis. This result may be explained by the findings of Tenneti *et al.* showing S-nitrosylation mediated by NO can inhibit the activation process of procaspases or inactivate caspases itself.^35^ Flow cytometry as well as TUNEL assay could not show rising cell numbers undergoing apoptosis exhibiting annexin V on the surface and fragmented DNA in nuclei in U87, LN229 and primary IC glioma cells. Nonetheless, we can observe the cleavage of PARP1 into a fragment size of 89 kDa that is normally linked to apoptosis.^36^ Inactivation by cleaved caspase 3 was not observed. Some agents, for example ethanol or H_2_O_2_, are known to induce necrotic processes with cleavage of PARP1 in the fragment size of 89 and 50 kDa or additional fragments up to 35 kDa. We did not observe this in glioma cells by western blot analysis.^37^ It is described for alkylating agents that PARP1-mediated necrosis can be induced at the same time as depletion of intracellular ATP.^18^ The apoptotic incident requires plenty of energy for synthesis of proteins and enzymatic activity for DNA repair and maintenance of cell integrity.^38^ During the process of apoptosis, mitochondria stop providing ATP as the molecular energy source and glycolysis takes over.^39, 40^ In case of necrosis mitochondria stop producing energy and cells cannot prevent the shutdown of metabolism.^29, 41^ In this study, the alteration of the intracellular ATP level was investigated luminometrically and, as expected, the etoposide-treated apoptotic cells produced more ATP than the untreated control cells whereas necrotic cells induced by H_2_O_2_ stop providing ATP. The JS-K treated cells approach the value of the necrotic control in a dose- and time-dependent manner down to 11% of untreated controls. This aspect can be interpreted as a necrotic process with dose-dependent depletion of ATP; in addition to a small population of cells undergoing apoptosis, they concurrently maintain the ATP level by induction of glycolysis. Or it is just an exclusive necrotic event that is not that fatal as the necrotic control but nonetheless dose-dependent to JS-K exposure. In contrast, it is known for tumour cells in a confined environment such as in brain tumours that they can maintain their intracellular energy level with a mixture of glycolysis and oxidative phosphorylation termed aerobic glycolysis.^42^ Since NO is known for its ability to activate soluble guanylyl cyclase we investigated the intracellular level of cGMP after JS-K treatment for an inference to the obvious non-apoptotic pathway.^43^ Shen *et al* could show that cGMP protects endothelial cells against apoptosis mediated by NO.^44^ The way cells prevent apoptosis by cGMP signalling was investigated by Ha *et al.*^27^ They could show that inhibition of caspase 3 and phosphorylation of the Akt-survival pathway by cGMP pathway is responsible for the downregulation of apoptosis in adrenal gland cells. Kim *et al* demonstrated direct inhibition of caspase 3 activation through S-nitrosylation of the enzyme mediated by NO.^45^ In previous publications our group could show the effect of JS-K on cGMP pathway in U87 cells. It could be proved by guanylyl cyclase-inhibitor that NO has direct impact on cGMP in GBM cells.^46^ In this study we explored the direct connection between upregulated cGMP level and dose-dependent phosphorylation of the proteinkinase Akt by JS-K. Since the increase of cGMP in also observed in necrotic cells exposed to H_2_O_2_ we conclude that the activation of cGMP is linked to necrotic mechanisms induced by NO. Apoptosis does not have any effect on cGMP in our experiments. Therefore we assume that NO is preventing apoptosis in GBM cells by inhibition of caspase 3 cleavage and activation of survival signalling. In addition to the cellular processes, the morphology of the glioma cells changes time- and dose-dependently after exposure to JS-K. The nuclei of the GBM cells were either swollen or multifragmented with an enlarged cytoplasm. This phenomenon is called MC, which is described by morphological criteria but not defined by a specific molecular pattern. MC is described as incomplete mitosis particularly after DNA damage leading to endopolyploidy, which finally leads to apoptosis or necrosis.^23, 25^ Cells resistant to apoptosis are selected in the process of DNA repair and may show morphological characteristics of MC or survive.^21^ For discrimination of apoptosis, Nabha *et al* demonstrate in experiments with caspase inhibitors that apoptosis has no influence on the morphological changes and the viability of cells.^24^ Since tumour cells are frequently deficient in cell cycle checkpoints, they are more susceptible to DNA damage and MC induced by agents such as JS-K.^25^ The morphological changes we observe in GBM cells treated with NO are those attributed to MC. MC appears earlier than PARP1 cleavage and the reduction of viability as shown in the MTT assay. Therefore, we assume that high-dose JS-K results in the induction of necrosis rather than apoptosis in glioma cells. Even if necrosis is not the favoured mechanisms undergoing cell death in human organism, MC induced by NO is a promising outcome for the tumour environment and progression in the treatment of GBM. The alternative pathway of MC can be useful for anti-cancer strategies to circumvent anti-apoptotic and drug-resistance mechanisms. It would be interesting to show in further experiments how glioma cells respond to NO under hypoxic conditions typical for tumour microenvironment to confirm the therapeutic role of NO-induced MC in glioblastoma.

## Conclusions

In this study we demonstrate for the first time that MC is the major cell death mechanism in glioblastoma caused by the NO donor JS-K *in vitro*. With this treatment we show that apoptosis plays a minor role in cell death in malignant gliomas possibly secondary to inhibition of caspase signalling pathways. Most GBM cells undergo cell death by necrosis resulting from induction of MC. Our results suggest a putative benefit of JS-K as an adjuvant in the multimodal therapy of GBM allowing the circumvention of anti-apoptotic mechanisms by the induction of MC leading to necrosis.

## Materials and Methods

### Cell culture

Established human glioma cell lines U87MG and LN229 and the primary glioblastoma cell line IC were cultured in Dulbecco's modified Eagle medium containing 10% fetal bovine serum and 0.5% ciprofloxacin at 37 °C in a humidified atmosphere containing 5% CO_2_. The primary cell line was established from surgical specimen from a patient (IC) with GBM. Retrieval and scientific analysis of patient-derived tissue was approved by the local ethics committee under protocol 100020/09. The NO donor JS-K [*O*2-(2,4-dinitrophenyl)1 [(4-ethoxycarbonyl)piperazin-1-yl]diazen-1-ium-1,2-diolate] was synthesized as described earlier.^47^ Cells were exposed to JS-K in concentrations between 1 and 25 *μ*M (stock solution 5.2 mM in DMSO) for 48 h and 72 h when they reached 70–80% confluence. The final concentration of DMSO was not higher than 0.48% (25 *μ*M).

### MTT assay

Cell viability was determined by MTT assay. 10^4^ cells were grown in 96 well plates with complete Dulbecco's modified Eagle medium and treated with 1–25 *μ*M JS-K for 48 or 72 h. MTT assay was performed as described before.^13^ Absorbance at 570 nm was measured with Tecan Infinite200 (Tecan, Männedorf, Switzerland). Percentages were calculated relative to viability of untreated controls set to 100%.

### Western blotting

Whole-cell lysates of JS-K treated cells were prepared from U87, LN229 und primary IC glioma cells with lysis buffer (50 mM Tris-HCL, pH 7.5, 150 mM NaCl, 1 mM EDTA, 0.5% sodium deoxycholate, 0.05% sodium dodecyl sulfate, 1% igepal containing protease inhibitor cocktail; Roche, Mannheim, Germany). Untreated as well as DMSO controls (0.28%) were performed. Equal amounts of protein (20–30 *μ*g) were applied on 12% SDS-polyacrylamide gels and electrophoresed (BioRad, Munich, Germany). Proteins were blotted on PVDF-membranes by wet blotting (BioRad). Epitopes were blocked with 5% non-fat milk in tris-buffered saline with 0.05% Tween-20 for 1 h at room temperature. Blots were incubated with primary antibodies anti-PARP1 (1:1000 Cell Signaling Technology, Inc., Danvers, MA, USA), anti-caspase 3, 8, 9 (1:500 Cell Signaling Technology, Inc.), anti-GAPDH (1:10000 abcam, Cambridge, UK) overnight at 4 °C. After incubation with secondary antibodies goat anti-rabbit (PARP1, caspases) and goat anti-mouse (GAPDH) (Santa Cruz Biotechnology, Santa Cruz, CA, USA) for 1 h at room temperature proteins were visualized by enhanced chemiluminescence (PerkinElmer, Milano, Italy), quantified with ImageJ (National Institutes of Health (NIH), Bethesda, MD, USA) and compared by unpaired two-tailed Student's *t*-test.

### ATP determination

ATP levels of glioma cells were determined luminometrically after treatment with JS-K (1–15 *μ*M). Cells were lysed in 100 *μ*l lysis buffer (25 mM Tris-HCL, pH 7.5, 0.1% Triton-X) and frozen in liquid nitrogen for 30 s. Chemicals were used according to the manufacturer's protocol (ATP Determination Kit, Molecular Probes, Eugene, USA) with 10 *μ*l of each sample. Controls were performed for induction of apoptosis by application of 10 *μ*M etoposide for 4 h and for necrosis by 3 mM H_2_O_2_ for 4 h. Lysates of untreated and DMSO-treated cells were included as controls.

### Evaluation of intracellular cGMP

Enzyme immunoassay was performed to determine intracellular cGMP level of glioma cells exposed to JS-K for 48 h. Chemicals were used according to the instructions provided by the supplier (Cyclic GMP XP Assay Kit, Cell Signaling Technology, Inc.). Controls were performed for apoptosis by application of 10 *μ*M etoposide for 4 h and for necrosis by 3 mM H_2_O_2_ for 4 h. Lysates of untreated and DMSO-treated cells were included as controls. cGMP level was calculated to amount of protein.

### Flow cytometry

Flow cytometry was used to distinguish between apoptosis and necrosis with staining for annexin V (LifeTechnologies Alexa Fluor 647 #23204) and with PI. Supernatant of JS-K treated cells was centrifuged for 5 min at 1500 rpm, adherent cells were trypsinized and centrifuged. Cell pellets were pooled and washed with PBS. Cells were stained with annexin V (1:200) and PI (1:46) in 500 *μ*l binding buffer (10 mM HEPES, pH 7.4, 140 mM NaCl, 2.5 mM CaCl_2_, 0.1% BSA) for 15 min on ice. 2 × 10^4^ cells were acquired by flow cytometry at FACSCalibur (Beckman Coulter, Brea, CA, USA) and the presence of viable cells (annexin V-and PI-negative), apoptotic cells (annexin V-positive, PI-negative) and necrotic cells (annexin V- and/or PI-positive) were analysed using Kaluza Analysis Software (Beckman Coulter).

### TUNEL assay

Glioma cell lines U87, LN229 and primary cells IC were cultured on 12 mm coverslips and treated with JS-K in concentrations of 1, 5 and 10 *μ*M and DMSO as additional control. Cells were stained according to the manufacturer's protocol (ApopTag Peroxidase *In Situ* Apoptosis Detection Kit, Millipore, Temecula, CA, USA). The 3, 3'-diaminobenzidine (DAB)-staining for apoptotic cells was performed according to the manufacturer's instruction (DAB Peroxidase Substrate Kit, Vector Laboratories, Burlingame, CA, USA) for 20–60 s and washed three times with ddH_2_O. The cells were mounted with Entellan (Merck, Darmstadt, Germany) and visualized by microscopy (Zeiss, Oberkochen, Germany). The optical criteria for MC in this experiment were multifragmented nuclei and extended soma or broad nuclei compared to untreated cells according to the description by Roninson *et al.*^21^

### Statistical analysis

All experiments were performed in triplicates. Data are shown as mean±s.d. Data were compared using an unpaired two-tailed Student's *t*-test, *P*<0.05 was considered statistically significant.

## Abbreviations

ATP: adenosine triphosphate
BBB: blood-brain-barrier
cGMP: cyclic guanosine 3',5'-monophosphate
CO_2_: carbon dioxide
DAB: 3,3'-diaminobenzidine
ddH_2_O: double-distilled water
DMEM: Dulbecco's modified Eagle medium
DMSO: dimethyl sulfoxide
GAPDH: glyceraldehyde 3-phosphate dehydrogenase
ECL: enhanced chemiluminescence
GBM: glioblastoma multiforme
GST: glutathione S-transferase
H: hour
H_2_O_2_: hydrogen peroxide
JS-K: (O2-(2,4-dinitrophenyl) 1-[(4-ethoxycarbonyl)piperazin- 1-yl]diazen-1-ium-1,2-diolate)
kDa: kilodalton
MAPK: mitogen-activated protein kinase
MC: mitotic catastrophe
min: minute
MTT: 3-(4,5-dimethylthiazol-2-yl)-2,5-diphenyltetrazolium bromide
mM: milimolar
mm: millimeter
*μ*g: microgram
*μ*l: microliter
*μ*M: micromolar
*μ*m: micrometer
nm: nanometer
NO: nitric oxide
PARP1: Poly(ADP-ribose)-Polymerase 1
PBS: phosphate-buffered saline
PI: propidium iodide
PVDF: polyvinylidene fluoride
Rpm: rounds per minute
RT: room temperature
SD: standard deviation
SDS: sodium dodecyl sulfate
sec: seconds
TUNEL: terminal deoxynucleotidyl transferase dUTP nick end labeling
